# Comprehensive Investigation into the Antioxidant Phytochemicals and Molecular Mechanisms of the Genus *Ottelia*: An Integrated Metabolomic and Network Pharmacology Approach

**DOI:** 10.3390/ijms27094071

**Published:** 2026-05-01

**Authors:** Yanan Liu, Chenghao Zhu, Han Zhang, Yishang Yang, Fengluan Tang

**Affiliations:** Guangxi Institute of Botany, Chinese Academy of Sciences, Guilin 541006, China; medicallyn@163.com (Y.L.); zch0522@gxib.cn (C.Z.); yangyishan0113@163.com (Y.Y.)

**Keywords:** *Ottelia acuminata*, *O. acuminata* var. *jingxiensis*, *O. fengshanensis*, *O. guanyangensis*, *O. alismoides*, antioxidant, phytochemicals, molecular mechanisms

## Abstract

As endemic or regionally distinctive taxa in China, *Ottelia* species exhibit notable ecological adaptability as well as potential nutritional and medicinal value. Nevertheless, a comprehensive characterization and comparative analysis of the chemical constituents across this genus remain lacking, particularly for *O. acuminata* var. *jingxiensis*, *O. fengshanensis*, and *O. guanyangensis.* This knowledge gap has hindered the systematic exploitation and value-added utilization of *Ottelia* resources. In the present study, five *Ottelia* taxa—*O. acuminata*, *O. acuminata* var. *jingxiensis*, *O. fengshanensis*, *O. guanyangensis*, and *O. alismoides—*were investigated. By integrating widely targeted metabolomics, network pharmacology, and in vitro experimental validation, we identified Rivularin, Tenaxin I, Sinensetin, 8-Methoxyapigenin, Chrysoeriol, Hispidulin, Genkwanin, 5,2′-Dihydroxy-7,8-dimethoxyflavone, Kumatakenin, and Pectolinarigenin as key antioxidant constituents in the Genus *Ottelia*. Network-based analyses further indicated that these compounds predominantly act on PTGS1/2 and AR, and may mediate antioxidant activity primarily through the PI3K–Akt signaling pathway and pathways associated with EGFR tyrosine kinase inhibitor resistance. Collectively, these findings provide a scientific basis for the further development, functional evaluation, and sustainable utilization of the Genus *Ottelia*.

## 1. Introduction

The genus *Ottelia* (Hydrocharitaceae) comprises approximately 40 species of perennial, fully submerged aquatic herbs distributed predominantly in tropical and subtropical regions [1]. In China, the genus is represented by 10 species, most of which occur in the heterogeneous karst landscapes of the southeastern and southwestern provinces [2]. These aquatic macrophytes are not merely ornamental elements of freshwater ecosystems; rather, they function as sensitive ecological indicators. Their pronounced responsiveness to fluctuations in water quality renders them valuable for environmental monitoring and for sustaining the stability of aquatic plant communities [3]. In addition to their ecological significance, several *Ottelia* species possess a long-standing ethnobotanical history among ethnic communities in the Yunnan-Guizhou province, where they have been traditionally consumed as nutrient-rich vegetables and used as medicinal resources for centuries [4].

*Ottelia acuminata* (*OC*)—locally known as “dragon-claw vegetable,” “seaweed,” and “water poplar”—is a Chinese endemic species occurring mainly in Yunnan, Guizhou, and Guangxi. It has long been used by local ethnic communities as both a traditional vegetable and a medicinal resource [5]. The tender leaves are typically blanched in boiling water to reduce astringency and then consumed stir-fried, as a cold dish, or in soups. The cooked leaves are described as having a light and refreshing taste. Consequently, *O. acuminata* is a commonly utilized edible and medicinal plant in local diets [4,6]. *Ottelia acuminata* var. *jingxiensis* (*JXOC*) is a regional variety primarily distributed in Jingxi, Guangxi. Its culinary use is comparable to that of *O. acuminata*, and its tender leaves are reportedly rich in vitamin C, dietary fiber, and multiple minerals. However, owing to its restricted geographic range, its consumption remains largely confined to areas near its native habitats [3]. *Ottelia fengshanensis* (*FSOF*) and *Ottelia guanyangensis* (*GYOF*) are also endemic and rare species in Guangxi. The tender leaves of both species can be harvested and consumed as wild vegetables; following simple preparation, they exhibit a mildly sweet flavor and provide appreciable nutritional value. However, owing to their small population sizes and stringent habitat requirements, these species have not been adopted for large-scale food use [1,7]. In contrast, *Ottelia alismoides* (*OA*) exhibits a broad distribution range. Its seedlings and young leaves are edible and are characterized by a crisp, tender texture, making them suitable for stir-frying and soups [8]. To date, many researchers have undertaken systematic rescue surveys and implemented conservation measures for this genus. These plant resources have been effectively protected and reproduced, and their yields have been steadily increasing [9,10,11,12,13]. However, the research on their medicinal value is mainly limited to ethnic botany and historical records, while comprehensive and systematic bioactivity studies are still relatively lacking. Therefore, the medicinal potential of the genus *Ottelia* as a functional food still requires further in-depth exploration. The genus Ottelia can all be processed simply for consumption. In the field of research on medicinal and edible plants, antioxidant activity is regarded as the primary research direction [14]. This is determined jointly by the transformation of the modern chronic disease spectrum and the natural characteristics of phytochemicals. Oxidative stress is the common pathological and physiological basis of most chronic diseases [15], and during the consumption of these plants, they engage in long-term, mild, and multi-targeted regulation of the body to prevent the occurrence and development of diseases. If a medicinal and edible plant does not have even basic antioxidant activity, then its performance in complex anti-inflammatory, anti-tumor, and anti-aging aspects is usually not outstanding [16]. Therefore, based on the above viewpoints, this study mainly focuses on the antioxidant activity of these plants.

Widely targeted metabolomics integrates the strengths of targeted and untargeted approaches, enabling broad-coverage qualitative profiling alongside high-sensitivity quantitative determination of metabolites [17]. This strategy can conduct comprehensive and systematic comparative analysis of metabolites from different species [18,19]. This study employed a widely targeted metabolomics approach to conduct pairwise comparative analyses among five species. Shared differentially accumulated metabolites were identified and leveraged to enhance the precision of chemical component annotation in these plants. Subsequently, network pharmacology was applied to systematically screen for primary antioxidant-active constituents [20], followed by experimental validation. Integrating metabolomics and network pharmacology enabled the construction of a comprehensive antioxidant-related metabolite landscape. By identifying core bioactive constituents and their putative molecular targets, this work establishes a robust foundation for advancing these traditional resources toward evidence-based functional food development.

## 2. Results

### 2.1. Metabolomics

**Figure 1:**
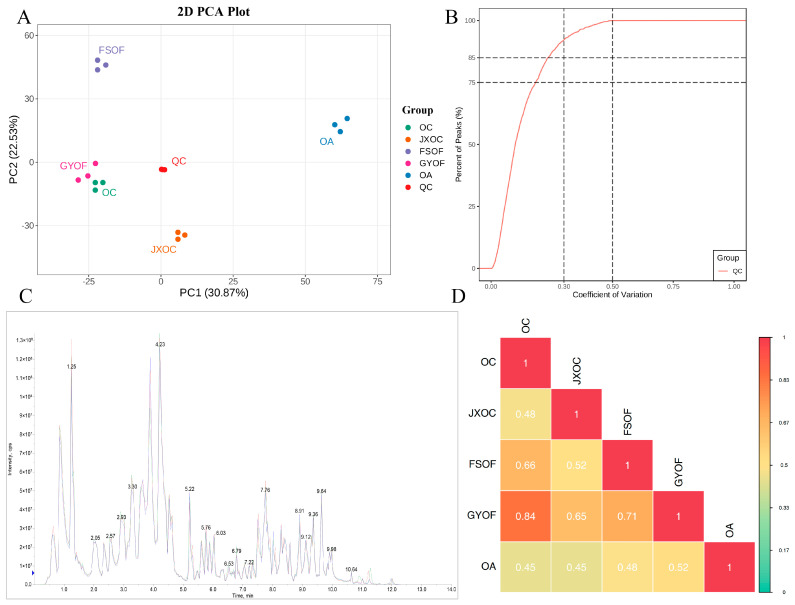
Sample quality control information. (**A**) PCA analysis. (**B**) Distribution map of coefficient of variation (CV) for each group of samples. (**C**) Total ion chromatogram of mass spectrometry analysis for mixed samples. (**D**) Correlation plot among experimental samples.

The PCA results of metabolomic analysis showed effective separation among various samples (differences exist between samples, Figure 1A). The proportion of substances with CV values less than 0.3 in the QC samples was higher than 75%, indicating that the experimental data were very stable (Figure 1B). The total ion chromatogram of the QC sample for mass spectrometry analysis is shown in Figure 1C (positive ion mode). The results showed high overlap in the curves of total ion flow detected from metabolites, indicating consistent retention time and peak intensity. This suggests good instrument stability during mass spectrometry analysis of the same sample at different times. Through correlation analysis between samples (Figure 1D), it was found that the correlation between *OC* and *GYOF* was 0.84, indicating a strong correlation between the two.

**Figure 2:**
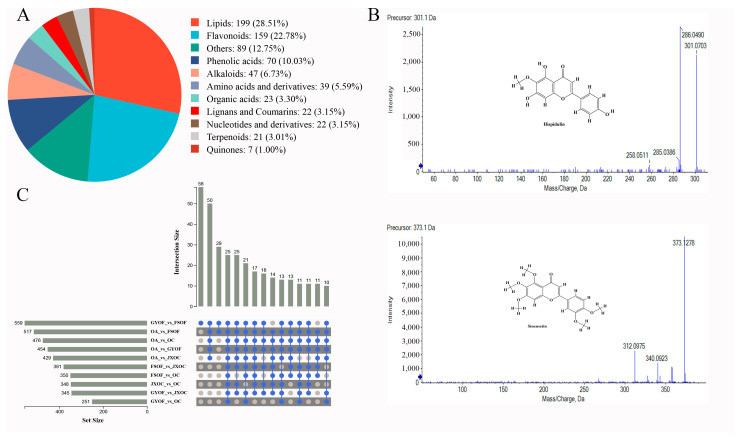
Secondary spectrum of some identified chemical components. (**A**) Types of level-1 metabolites. (**B**) Secondary mass spectra of identified compounds. (**C**) Quantity of differential metabolites in pairwise species comparisons, the blue sphere indicates the presence.

A total of 2560 compounds were annotated across five *Ottelia* species. Among the identified metabolites, trans-caffeic acid, 6,7,8-tetrahydroxy-5-methoxyflavone, scutevulin, pratensein, diosmetin, argemonine, 5,6,7-trihydroxy-8-methoxyflavone, and curculigine were found to be exclusive to *OA*, as they remained unannotated in all other sampled species (Table S1). Pairwise comparisons among species were subsequently performed to screen and consolidate differentially accumulated metabolites, yielding 698 metabolites annotated with confidence level 1 (compounds confirmed by reference to standard samples under the same analytical conditions). Among these level-1 metabolites, lipids, flavonoids, phenolic acids, and alkaloids were relatively abundant (Figure 2A), accounting for 28.51%, 22.78%, 10.03%, and 6.73% of the annotated metabolites, respectively. Detailed metabolite information is provided in Supplementary Table S2. Bioactivity-related screening of the 698 annotated metabolites indicated that 125 compounds met the predefined oral bioavailability (OB), drug-likeness (DL), and Druglikeness criteria (Table S3). Representative compounds included laurocapram, avicennol, aurantiamide, epiloliolide, and vasicinone (Figure 2B). Target prediction for these 125 candidate compounds yielded 424 compound–target interactions after integration and removal of redundant entries.

In addition, among the 698 chemical components, 58 components showed differences only between *GYOF* and *FSOF* (Figure 2C), mainly including Senkyunolide C, Methylillicinone F, Monopalmitin, 2-Palmitoylglycerol, and Eicosadienoic acid, as detailed in Supplementary Table S4. There were 25 components that showed significant differences between every pair of species (Figure 2C), as shown in Table 1, mainly including Pectolinarigenin, Kaempferol-3-O-sambubioside, Gardenin D, naringenin 5-glucoside, and Choerospondin, which are mainly flavonoids and phenolic acids.

### 2.2. Antioxidant-Related Targets

Antioxidant-related targets were retrieved from the GeneCards database, and candidates were defined as genes annotated with antioxidant activity and a relevance score > 2.0 (the score located at the median). In total, 681 related targets were obtained (Table S5), including *NFE2L2* (NFE2 like BZIP transcription factor 2), *SOD1* (superoxide dismutase 1), *CAT* (catalase), *SOD2-OT1* (SOD2 overlapping transcript 1), and *ATOX1* (antioxidant 1 copper chaperone). These genes are directly or indirectly involved in oxidative stress, ROS metabolism, antioxidant defense, and disease states associated with oxidative damage. Among them, *SOD1* and *SOD2* convert superoxide anions into hydrogen peroxide, while *GPX1* and *GPX2* remove lipid peroxides. These genes are mainly antioxidant enzymes that eliminate free radicals and systems that maintain the redox balance within cells. *NOX1* and *NOX4* are members of the *NADPH* oxidase family and mainly participate in ROS metabolism. *NFE2L2* mainly participates in Oxidative Stress Regulation. And *APP*, *BACE1*, *CASP3*, *PTGS2*, and *VEGFA* are mainly related to Disease States Associated with Oxidative Injury.

### 2.3. Construction of the Active Compound–Antioxidant Target PPI Network

**Figure 3:**
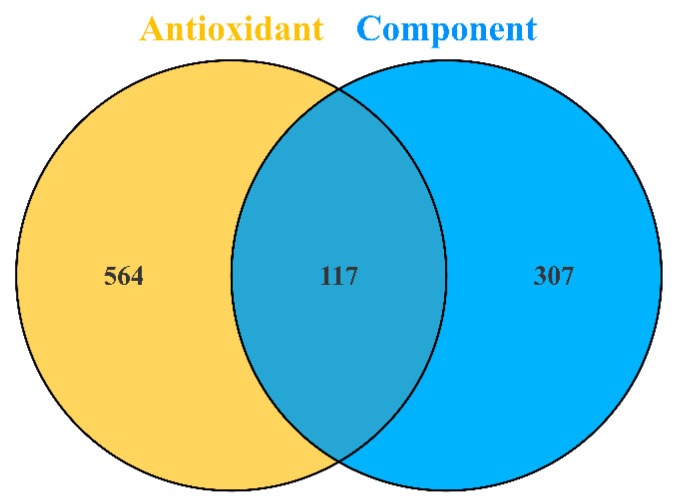
Component–antioxidant target information.

**Figure 4:**
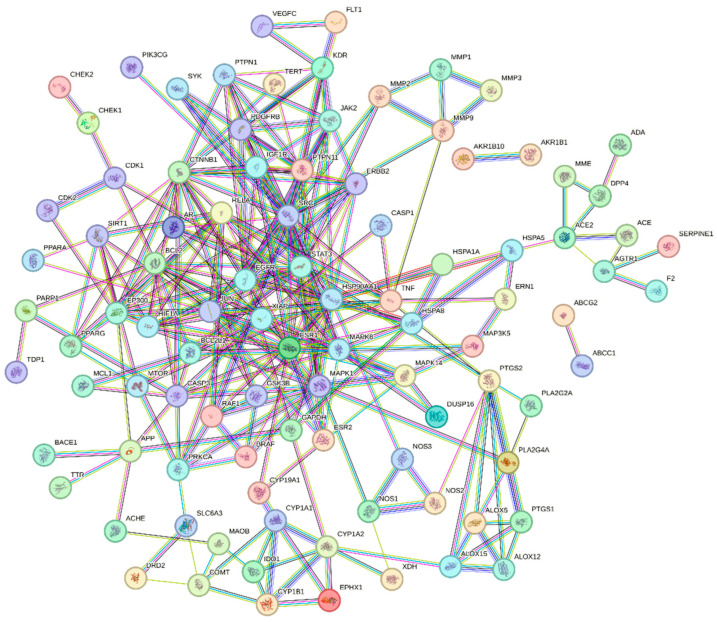
The 117-component–antioxidant target PPI network.

By intersecting the predicted targets of candidate compounds with antioxidant-related targets, 117 overlapping targets associated with antioxidant effects were identified (Figure 3). These targets were imported into the STRING v11.5 database to construct a protein–protein interaction (PPI) network under a high-confidence threshold (interaction score = 0.900) (Figure 4). Functional annotation indicated that the 117 targets were mainly involved in the cellular response to oxygen-containing compounds (Biological Process), enzyme binding (Molecular Function), and vesicles (Cellular Component).

**Figure 5:**
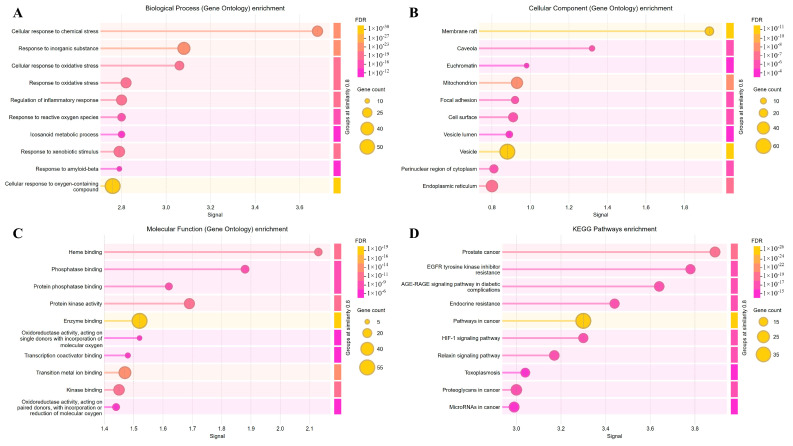
GO and KEGG analysis of 117 components and antioxidant targets. (**A**) Biosynthetic enrichment pathway. (**B**) Cellular component enrichment pathway. (**C**) Molecular function enrichment pathway. (**D**) KEGG pathway enrichment analysis.

Pathway enrichment analysis showed significant overrepresentation of pathways related to prostate cancer, EGFR tyrosine kinase inhibitor resistance, the AGE–RAGE signaling pathway in diabetic complications, endocrine resistance, pathways in cancer, the HIF-1 signaling pathway, the relaxin signaling pathway, and toxoplasmosis (Figure 5). Further network topology and module detection identified seven densely connected clusters. Proteins within the major modules included *TNF*, *RELA*, *MMP2*, *HIF1A*, *MAPK8*, *EGFR*, *BCL2*, *STAT3*, *SIRT1*, *CTNNB1*, *MMP9*, *ERBB2*, *PTGS2*, *EP300*, *GAPDH*, *JUN*, *SRC*, *ESR1*, and *MAPK1* (Table 2 and Table 3).

Traditional antioxidants (such as vitamin C) are direct scavengers of free radicals. However, network pharmacology reveals the gene regulatory network. Enriched in pathways such as prostate cancer and EGFR tyrosine kinase inhibitor resistance, this indicated that the chemical components in the *Ottelia* plants may mainly play the role of signaling regulatory molecules. Rather than merely neutralizing reactive oxygen species, these compounds appear to exert their antioxidant effects indirectly. They bind to cell surface receptors (e.g., EGFR), modulate downstream kinase cascades, and ultimately activate endogenous cellular defense mechanisms—most notably the Nrf2 signaling pathway—which upregulates the expression of antioxidant enzymes such as superoxide dismutase (SOD) and catalase (CAT).

### 2.4. Construction of the Compound–Target–Pathway Network

**Figure 6:**
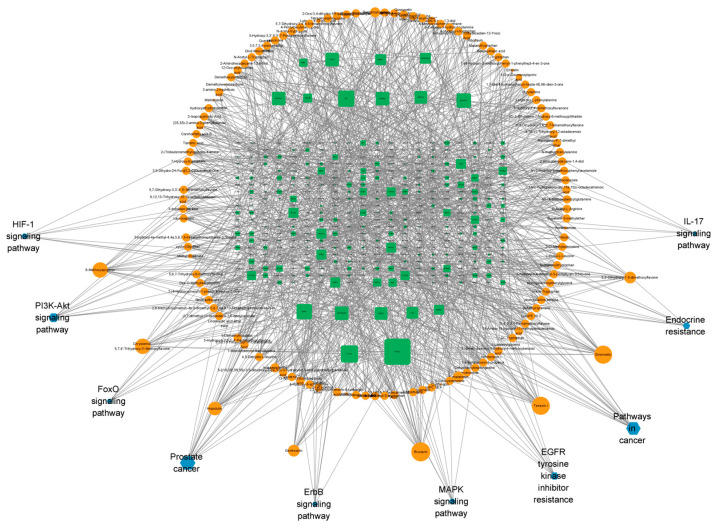
Network of 125 components, antioxidant targets, and action pathways.

A compound–antioxidant target network was constructed in Cytoscape v3.9.1 (Figure 6). Network topological properties were quantified using the built-in NetworkAnalyzer module, and key antioxidant-related compounds and core targets were identified (Table 3). Topological analysis indicated that rivularin exhibited a high level of connectivity (degree = 22), with betweenness centrality = 0.0431733 and closeness centrality = 0.2710280, suggesting that rivularin is a principal antioxidant-associated constituent among the five *Ottelia* taxa. Tenaxin I ranked second (degree = 21, betweenness centrality = 0.0295569, closeness centrality = 0.2663122). Among the targets, PTGS2 showed the highest network centrality (degree = 30, betweenness centrality = 0.0868645, closeness centrality = 0.2821300), followed by PTGS1 (degree = 20), AR (degree = 19), and DPP4 (degree = 18). Pathway-level mapping highlighted prostate cancer, pathways in cancer, the PI3K–Akt signaling pathway, and EGFR tyrosine kinase inhibitor resistance as the predominant enriched pathways within the network.

### 2.5. Molecular Docking Results 

**Figure 7:**
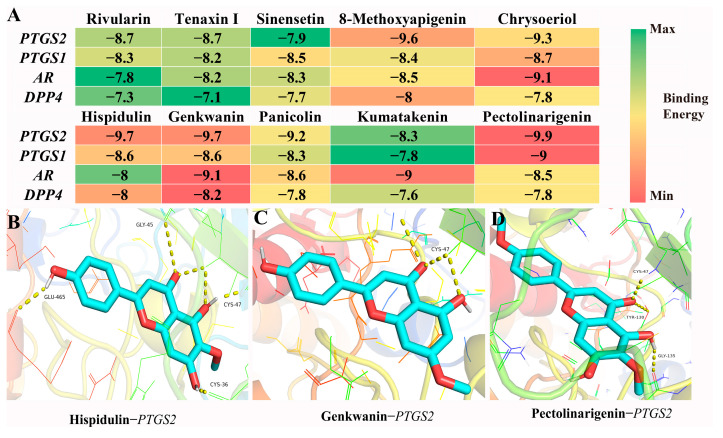
Visualization of the docking results between ten components and four targets. (**A**) Docking scores for 10 compounds with 4 targets; (**B**–**D**) represent the visualization of docking results of Hispidulin, Genkwanin, and Pectolinarigenin with *PTGS2*.

The 10 compounds in Table 3 were subjected to molecular docking with the 4 targets (*PTGS2*, *PTGS1*, *AR*, *DPP4*). The 4 targets were *PTGS2* (PDB ID: 5F19), *PTGS1* (PDB ID: 6Y3C), *AR* (PDB ID: 1XOW), and *DPP4* (PDB ID: 9LBT). The detailed docking information can be found in Table S6. The docking results showed that the binding energies of the three ligands, Hispidulin, Genkwanin, and Pectolinarigenin, to *PTGS2* were relatively small (−9.7 kcal/mol, −9.7 kcal/mol, and −9.9 kcal/mol, respectively). Among them, Hispidulin formed 6 hydrogen bonds with *PTGS2* through GLU-465, GLY-45, CYS-47, and CYS-36; Genkwanin formed 3 hydrogen bonds with *PTGS2* through CYS-47; and Pectolinarigenin formed 3 hydrogen bonds with *PTGS2* through CYS-47, TYR-130, and GLY-135. The interactions of these three groups were relatively strong (Figure 7B–D). In summary, the binding energies of *PTGS2* to each component were relatively low, making it the main receptor for the antioxidant activity of the chemical components of the genus *Ottelia*.

### 2.6. In Vitro Antioxidant Activity

**Figure 8:**
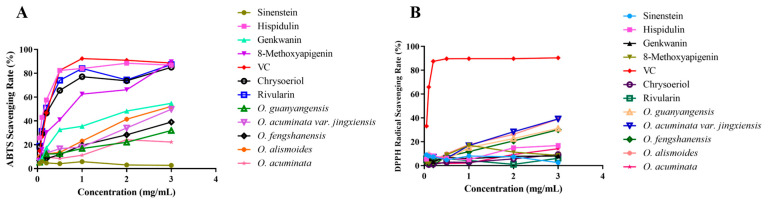
Comparison of antioxidant activities of five Ottelia ethanol extractions and the individual components. (**A**,**B**) The scavenging rates of ABTS and DPPH for each sample.

The antioxidant activity of the chemical components in Table 3 was tested using VC, *Ottelia acuminata*, *O. acuminata* var. *jingxiensis*, *O. fengshanensis*, *O. guanyangensis*, and *O. alismoides* as controls (Figure 8). The results showed that the 3 mg/mL extracts of *O. acuminata* var. *jingxiensis* and *O. alismoides* had clearance rates of 39.13% and 39.03% for DPPH free radicals, and 49.77% and 52.18% for ABTS free radicals. Among the five *Ottelia* extracts, they performed well. While 3 mg/mL of Hispidulin had clearance rates of 16.78% and 86.83% for DPPH and ABTS free radicals, respectively, this result was consistent with the results of molecular docking. Additionally, 3 mg/mL of 8-Methoxyapigenin, Rivularin, and Chrysoeriol had clearance rates of 89.59%, 87.41%, and 85.02% for ABTS free radicals, respectively, but the ability of these three compounds to clear DPPH free radicals was relatively weak.

### 2.7. RT-qPCR

**Figure 9:**
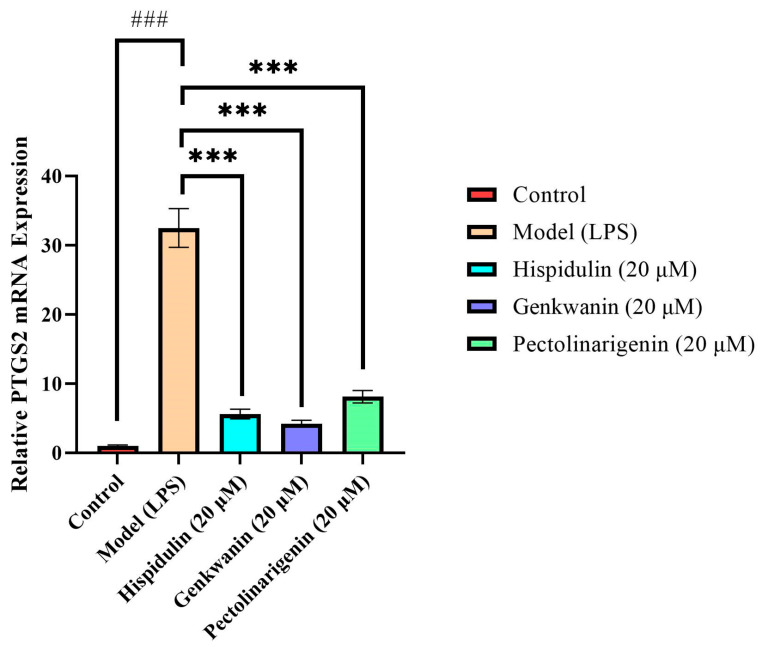
Effects of Hispidulin, Genkwanin, and Pectolinarigenin on *PTGS2* Expression. ***/### indicates significant differences (*p* < 0.001).

Compared with the Control group, the expression level of *PTGS2* mRNA in the Model group (stimulated by LPS) increased sharply by approximately 32.5 times (*p* < 0.001). This indicated that the inflammation model was successfully established and the transcriptional level was strongly activated. After adding the three compounds, the mRNA level of *PTGS2* showed a highly significant decrease (*p* < 0.001). At the same 20 μM concentration, Genkwanin exhibited the strongest gene transcriptional inhibitory activity (relative expression level dropped to 4.29, with an inhibition rate of 86.8%), followed by Hispidulin and Pectolinarigenin (Figure 9).

## 3. Discussion

As representative aquatic angiosperms, *Ottelia* species occupy a distinctive position within the ethnomedicinal systems of Southwest China, particularly in Yunnan and Guizhou. *Ottelia acuminata* and *O. alismoides* are valued not only as traditional vegetables but also as ethnomedicinal resources that are traditionally used for heat-clearing, diuretic, and antitussive purposes [5,21]. In the present study, we systematically profiled the chemical composition of five *Ottelia* taxa and found that lipids, flavonoids, phenolic acids, and alkaloids constitute the major metabolite classes, accounting for 28.51%, 22.78%, 10.03%, and 6.73% of the annotated metabolites, respectively. The predominance of lipids (28.51%) is likely linked to the ecological and physiological adaptations of *Ottelia* as submerged aquatic plants. Previous studies have reported that *Ottelia* inflorescences and leaves are enriched in unsaturated fatty acids (UFAs), with UFA/total fatty acid (TFA) ratios frequently exceeding 50%. These lipid constituents not only serve as energy reserves but also contribute to maintaining membrane fluidity and enhancing tolerance to aquatic environmental stress. Notably, several glyceride-type metabolites identified here, including 1-monolinolenoyl-rac-glycerol and 1-linoleoylglycerol, exhibited high predicted oral bioavailability (OB) and drug-likeness (DL), suggesting potential bioaccessibility and a capacity to modulate lipid-related metabolic processes following dietary intake. Flavonoids constituted 22.78% of the metabolite set, consistent with earlier reports identifying 26 flavonoid constituents—predominantly luteolin derivatives and quercetin glycosides—in *O. acuminata* [5]. Within ethnopharmacological practice, flavonoids are often regarded as key material bases underlying the purported effects of “heat-clearing and detoxification” and “promoting diuresis and alleviating edema”. Structurally, the polyhydroxylated flavonoid scaffold confers strong free-radical scavenging capacity, which has been corroborated by in vitro antioxidant assays [22]. Although phenolic acids (10.03%) and alkaloids (6.73%) accounted for smaller proportions, their biological relevance should not be underestimated. Phenolic acids, including chlorogenic acid and caffeoyl malic acid, are broadly distributed across *Ottelia* tissues and have been associated with enzyme inhibitory activity and protection against DNA damage. The occurrence of alkaloids may also be linked to the traditional use of *Ottelia* in managing conditions such as cancer and tuberculosis, warranting further mechanistic and pharmacological investigation.

Oral bioavailability (OB) and drug-likeness (DL) are key determinants of whether a bioactive constituent can be advanced as a therapeutic candidate or incorporated into functional foods. In this study, multiple metabolites with relatively high predicted OB and DL values—including 1-Monolinolenoyl-Rac-Glycerol, 1-Linoleoylglycerol, Aurantiamide, Riboflavin, Demethylwedelolactone, 8-Methoxyapigenin, Pectolinarigenin, Complanatuside, Tenaxin I, and Rivularin—may represent a core set of candidate molecules underlying the putative bioactivities of *Ottelia* spp. Aurantiamide, a nitrogen-containing compound, has been reported to exhibit anti-inflammatory and immunomodulatory activities in ethnomedicinal contexts. However, toxicological assessments have suggested that aurantiamide may act as a potential hERG channel inhibitor, highlighting the importance of evaluating possible cardiotoxicity risks prior to application. Riboflavin, an essential micronutrient, contributes to endogenous antioxidant defense through its role in redox metabolism, and its high predicted OB supports the value of *Ottelia* extracts as a source of dietary vitamins. Demethylwedelolactone is a bioactive anti-inflammatory compound that is reported to act, at least in part, via modulation of the NF-κB signaling pathway. Its favorable DL value may indicate desirable physicochemical properties for intestinal absorption and stability, providing a plausible molecular basis for traditional claims related to cough relief and asthma alleviation in *Ottelia*-based remedies. In addition, flavonoids such as pectolinarigenin and tenaxin I have been shown in contemporary pharmacological studies to induce tumor cell apoptosis and inhibit angiogenesis, which is broadly concordant with the traditional use of *O. alismoides* in anti-tumor therapy.

In this study, we explored the molecular basis underlying the putative regulatory effects of *Ottelia* spp. by constructing an active compound–antioxidant target PPI network. Gene Ontology (GO) enrichment indicated that the overlapping targets were mainly associated with the cellular response to oxygen-containing compounds, enzyme binding, and vesicle-related cellular components. The Biological Process term “cellular response to oxygen-containing compounds” suggests that *Ottelia* constituents may modulate intracellular redox homeostasis. Oxidative stress represents a shared pathological hallmark of multiple chronic disorders, including cancer and diabetic complications; thus, mitigation of reactive oxygen species (ROS) may attenuate oxidative damage to cellular macromolecules. Enrichment of the Molecular Function category “enzyme binding” further implies that these bioactive molecules may influence enzyme-mediated redox regulation, either by enhancing the activity of endogenous antioxidant enzymes (e.g., superoxide dismutases, SODs) or by suppressing pro-oxidant enzymes (e.g., cyclooxygenases, COXs). Within the PPI network, TNF, RELA, MMP2, HIF1A, MAPK8, and EGFR emerged as central nodes. These targets are not only implicated in oxidative stress responses but also act as pivotal regulators of inflammation and tumorigenesis. Notably, TNF and RELA are key components of the NF-κB signaling axis, which can be activated under oxidative stress to drive the transcription of pro-inflammatory mediators. Modulation of these targets by Ottelia constituents may therefore contribute to anti-inflammatory effects consistent with traditional “heat-clearing” claims. Oxidative stress can also promote matrix metalloproteinase (MMP) activation, facilitating extracellular matrix degradation and enhancing metastatic potential. Accordingly, suppression of MMP2 may represent one mechanistic avenue through which *Ottelia* exhibits anti-tumor potential. HIF1A, a master transcriptional regulator of hypoxic adaptation, is integral to the maintenance of the tumor microenvironment. In this context, hispidulin has been reported to influence cellular metabolism via the HIF-1 signaling pathway, thereby potentially constraining cancer cell survival.

KEGG pathway enrichment analysis indicated that *Ottelia* constituents were significantly associated with pathways including prostate cancer, EGFR tyrosine kinase inhibitor resistance, and the AGE-RAGE signaling pathway. Enrichment in the prostate cancer pathway offers a plausible mechanistic rationale for traditional medicinal applications. Prostatic tissue is particularly susceptible to oxidative injury, and oxidative stress may promote androgen receptor (AR) dysregulation through mutational events and/or aberrant overexpression. In this study, AR emerged as a key target of *Ottelia*, and—together with the predicted regulatory capacity of core constituents such as rivularin and hispidulin—these findings suggest potential utility in prostate cancer prevention and/or as an adjunct to existing therapeutic strategies. Enrichment in the AGE–RAGE signaling pathway further underscores the potential relevance of Ottelia for metabolic disorders. Notably, DPP4 (dipeptidyl peptidase-4) was identified among the key targets. Pharmacological inhibition of DPP4 is known not only to improve glycemic control but also to alleviate oxidative stress-associated endothelial dysfunction, implying a mechanistically coherent link between *Ottelia* constituents and vascular protection. Finally, the PI3K–Akt signaling pathway—frequently regarded as an integrative hub for cell survival, metabolism, and stress responses—may mediate the observed multi-target features of *Ottelia*. Collectively, these results support a multi-component, multi-target, and multi-pathway mode of action consistent with the systems-level characteristics of ethnomedicinal interventions.

In this study, the antioxidant capacities of *Ottelia* extracts and representative monomeric constituents were quantitatively compared using DPPH and ABTS radical scavenging assays. At 3 mg/mL, the extract solutions of *O. acuminata* var. *jingxiensis* and *O. alismoides* showed ABTS radical scavenging rates of 49.77% and 52.18%, respectively. Evaluation of individual compounds further clarified the material basis underlying antioxidant activity. Hispidulin achieved an ABTS scavenging rate of 86.83% at 3 mg/mL, whereas its DPPH scavenging activity was only 16.78%, reflecting radical-dependent reactivity and the chemical selectivity of flavonoids toward different radical systems. Moreover, 8-methoxyapigenin, rivularin, and chrysoeriol exhibited consistently high ABTS scavenging activities (85–90%), supporting their designation as major antioxidant-active constituents.

Hispidulin, genkwanin, and pectolinarigenin significantly suppress *PTGS2* (COX-2) expression in a dose-dependent manner. In LPS-stimulated macrophage models, RT-qPCR and Western blot analyses consistently demonstrate that all three flavonoids markedly inhibit both *PTGS2* mRNA and protein levels [23,24]. Notably, pectolinarigenin has been shown to ameliorate acetaminophen-induced acute liver injury in vivo by mitigating oxidative stress and dampening the pro-inflammatory response [25]. Mechanistically, these compounds exert their anti-inflammatory effects primarily through transcriptional repression of *PTGS2*—mediated via modulation of upstream signaling pathways such as NF-κB or MAPK cascades—rather than direct enzymatic inhibition. Collectively, these findings corroborate and extend prior evidence supporting the role of dietary flavonoids as natural regulators of COX-2-dependent inflammation.

Based on these screening criteria, we propose hispidulin, 8-methoxyapigenin, rivularin, and chrysoeriol as candidate quality markers (Q-markers) for *Ottelia* species. Establishing Q-markers would facilitate the development of more rigorous quality standards for medicinal materials and provide a practical basis for subsequent functional food research and product development. In addition, the metabolite-class distribution observed here (with flavonoids accounting for ~22.78%) is broadly consistent with prior reports on *Ottelia* from Yunnan Province [5]. Mechanistically, this study extends previous work by applying network pharmacology to identify two putative non-traditional targets—AR and DPP4—thereby providing a more systems-level perspective than earlier investigations [26]. Nevertheless, the current validation is limited to in vitro assays, and in vivo pharmacokinetic and pharmacodynamic confirmation has not yet been performed. Future studies should therefore employ relevant animal models, such as prostate cancer xenograft models in mice or diabetic rat models [27], with particular emphasis on the effects of these proposed Q-markers on key pathway proteins [28] (e.g., p-Akt and HIF-1α).

## 4. Materials and Methods

### 4.1. Materials

Leaf tissues from five *Ottelia* were collected from the introduction garden of the Guangxi Institute of Botany. The sampling site is located at 110°17′ E, 25°01′ N (elevation 178 m) and is characterized by a canopy cover (density) > 0.5. Five species were identified by Tang Fengluan, a researcher from the Guangxi Institute of Botany, Chinese Academy of Sciences, based on traditional ecology. The specimens were stored in the Herbarium of the Biotechnology and Wild Rare Plant Conservation Center of the Guangxi Institute of Botany, with the following numbers: *Ottelia acuminata* (OC-002133), *Ottelia acuminata* var. *jingxiensis* (JXOC-002251), *Ottelia fengshanensis* (FSOF-002107), *Ottelia guanyangensis* (GYOF-002281), and *Ottelia alismoides* (OA-002233). For each taxon, three biological replicates were collected. For each replicate, 2 g of fresh leaf material was harvested and immediately stored at −80 °C until further analysis. Plant materials were subjected to vacuum freeze-drying using a freeze dryer (Scientz-100F). Subsequently, the dried samples were homogenized to a fine powder using a high-frequency oscillating mill (MM 400, Retsch) at 30 Hz for 1.5 min. An accurately weighed aliquot of 50 mg of the resulting powder was transferred into a microcentrifuge tube, followed by addition of 1200 μL of ice-cold 70% (*v*/*v*) methanol–water extraction solvent containing an internal standard and pre-cooled to −20 °C. The mixture was vortexed for 30 s every 30 min, repeated six times to ensure thorough extraction. Following centrifugation at 12,000 × g for 3 min at 4 °C, the supernatant was carefully collected, filtered through a 0.22-μm polytetrafluoroethylene (PTFE) syringe filter, and transferred into a chilled autosampler vial for subsequent UPLC–MS/MS analysis.

### 4.2. Plant Metabolomics

#### 4.2.1. UPLC Conditions

The sample extracts were analyzed using an UPLC-ESI-MS/MS system and Tandem mass spectrometry system [29]. The analytical conditions were as follows: UPLC: column, Agilent SB-C18 (1.8 μm, 2.1 mm × 100 mm). The mobile phase consisted of solvent A, pure water with 0.1% formic acid, and solvent B, acetonitrile with 0.1% formic acid. Sample measurements were performed with a gradient program that employed the starting conditions of 95% A, 5% B. Within 9 min, a linear gradient to 5% A, 95% B was programmed, and a composition of 5% A, 95% B was kept for 1 min. Subsequently, a composition of 95% A, 5.0% B was adjusted within 1.1 min and kept for 2.9 min. The flow velocity was set as 0.35 mL per minute. The column oven was set to 40 °C. The injection volume was 2 μL. The effluent was alternatively connected to an ESI-triple quadrupole-linear ion trap (QTRAP)-MS.

#### 4.2.2. ESI-Q TRAP-MS/MS

The ESI source operation parameters were as follows: source temperature 500 °C; ion spray voltage (IS) 5500 V (positive ion mode)/−4500 V (negative ion mode); ion source gas I (GSI), gas II (GSII), and curtain gas (CUR) were set at 50, 60, and 25 psi, respectively; the collision-activated dissociation (CAD) was high. QQQ scans were acquired as MRM experiments with collision gas (nitrogen) set to medium [30]. DP (declustering potential) and CE (collision energy) for individual MRM transitions were done with further DP and CE optimization. A specific set of MRM transitions were monitored for each period according to the metabolites eluted within this period.

#### 4.2.3. Qualitative Analysis

Metabolites were annotated using a self-constructed MWDB (MetWare Database) based on MS/MS spectral information. During data processing, isotopic peaks, adduct-related interferences (including K^+^, Na^+^, and NH_4_^+^ adducts), and overlapping signals arising from fragment ions of higher-molecular-weight compounds were excluded [31]. Differential metabolites were initially selected according to the variable importance in projection (VIP) values derived from the OPLS-DA model, with VIP > 1 as the inclusion criterion. The OPLS-DA was performed by Log2 conversion and centering within R (MetaboAnalystR, 1.0.1). Subsequently, univariate statistics were applied using *p*-values/false discovery rate (FDR) correction (for datasets with ≥2 biological replicates) and/or fold-change (FC) to further refine candidate metabolites. Metabolites with FC ≥ 2 or FC ≤ 0.5 were considered significantly differential.

### 4.3. Network Pharmacology Analysis

#### 4.3.1. Compound Screening

Compounds with identification confidence level 1 (Section 2.2) were subjected to oral bioavailability (OB) and drug-likeness (DL) evaluation using the Traditional Chinese Medicine Systems Pharmacology database (TCMSP, https://www.tcmsp-e.com/, accessed on 2 December 2025). Candidate compounds were retained based on the criteria OB > 30% and DL > 0.18. For compounds not available in TCMSP, canonical SMILES obtained from PubChem (https://pubchem.ncbi.nlm.nih.gov/, accessed on 2 December 2025) were used for ADME-related assessment and target prediction via SwissTargetPrediction (http://www.swisstargetprediction.ch/, accessed on 2 December 2025). Compounds meeting the OB/DL-related screening requirements were included, and their putative protein targets were predicted using SwissTargetPrediction. To standardize target nomenclature, all protein targets were mapped to official gene symbols using the UniProt database (https://www.uniprot.org, accessed on 2 December 2025) [32].

#### 4.3.2. Antioxidant Target Collection

Antioxidant-related targets were collected from the GeneCards database (https://www.genecards.org/, accessed on 2 December 2025) [33]. Targets with a relevance score > 2.0 (conduct a search in the Genecard database using “antioxidant” as the keyword, and identify targets with correlation scores exceeding the median as candidate targets) were considered candidate targets [34,35]. All targets were converted to gene symbols, and duplicates were removed.

#### 4.3.3. PPI Network Construction and Enrichment Analysis

Targets shared between the predicted compound targets and antioxidant-related targets were identified by intersection analysis (Table S7). The overlapping targets were then submitted to STRING (http://cn.string-db.org/, accessed on 2 December 2025) to construct a protein–protein interaction (PPI) network. During parameter configuration, a 0.9 confidence interval was chosen for the interaction score, while independent targets exhibiting no interaction were concealed. The resulting interaction network was imported into Cytoscape for visualization and topological editing. In parallel, the overlapping targets were uploaded to Metascape (http://metascape.org/gp/index.html, accessed on 2 December 2025), and the MCODE algorithm was applied to identify densely connected network modules. Functional enrichment analyses were subsequently performed to characterize the principal biological processes and signaling/metabolic pathways associated with this modulesprotein interaction database [36].

#### 4.3.4. Pathway Enrichment Analysis and Network Construction

Following intersection analysis between the predicted compound targets and antioxidant-related targets, the overlapping targets were subjected to pathway enrichment analysis and used to construct a compound–target interaction network. For network visualization, significantly enriched pathways were filtered using a threshold of *p* < 0.05. Cytoscape v3.9.1 was employed to generate the active compound–antioxidant target network, and its built-in analytical functions were used to compute key topological parameters, including degree, betweenness centrality, and closeness centrality. Core targets and the principal antioxidant-active compounds were then identified based on these network metrics.

#### 4.3.5. Molecular Docking

Key bioactive compounds and their 3D structures were selected from PubChem. Energy minimization was performed in ChemDraw 3D, after which the structures were converted to MOL2 format. The most influential targets within the PPI network were identified, and their corresponding UniProt accession numbers were retrieved from UniProt and used to obtain protein structures (PDB format) from the RCSB PDB database (https://www.rcsb.org/, accessed on 3 December 2025). Protein structures were prepared and optimized in PyMOL (v2.5.5). Subsequently, ligand (MOL2) and receptor (PDB) files were processed in AutoDockTools (https://autodock.scripps.edu/, accessed on 26 April 2026) and converted to PDBQT format for molecular docking. Docking was performed to estimate binding affinities, and binding energy scores were recorded. Finally, docking poses were visualized in PyMOL [37]. The selection of target proteins from the Uniprot database adheres to the principle of protein functionality, whereas the selection from the PDB primarily relies on the resolution of crystal structures obtained through X-ray diffraction techniques. In the preprocessing of target proteins, organic compounds and solvents were initially removed. The docking boxes were chosen to encompass the entire protein to the greatest extent possible, and Quercetin was employed as a reference ligand prior to docking each compound [38].

### 4.4. Antioxidant Assays

An aliquot of 3.0 mg of the reference standard was precisely weighed and transferred into a 1.5 mL light-shielded (amber) microcentrifuge tube. The substance was dissolved in 1.0 mL of anhydrous ethanol via ultrasonication to yield a primary stock solution at a concentration of 3.0 mg/mL. Subsequently, a series of working solutions (0.05, 0.10, 0.20, 0.50, 1.0, 2.0, and 3.0 mg/mL) were prepared through progressive dilution with anhydrous ethanol, maintaining a final volume of 700 μL for each concentration. All experimental groups, including the solvent blanks and positive controls (L-ascorbic acid at equivalent concentrations), were performed in triplicate to ensure reproducibility.

#### 4.4.1. DPPH Radical Scavenging Activity

DPPH radical (DPPH·) scavenging activity was evaluated using a commercial total antioxidant capacity assay kit, with L-ascorbic acid serving as the positive control. Appropriate volumes of the sample solution and control solution were accurately pipetted into the reaction system. Reagents were added strictly according to the manufacturer’s instructions, and absorbance was recorded at 515 nm [39].

$$\mathrm{DPPH}\cdot \;\mathrm{scavenging}\;\mathrm{rate}/\%=\frac{A_{\mathrm{NC}}-{\;A}_{\mathrm{Sample}}}{A_{\mathrm{NC}}}\times 100$$

Note: A_NC_ is the absorbance value measured after mixing the extraction solution with reagent 1; A_sample_ is the absorbance value after mixing the sample with reagent 1.

#### 4.4.2. ABTS Radical Scavenging Activity

ABTS radical (ABTS·) scavenging activity was assessed using a commercial ABTS· scavenging assay kit, with L-ascorbic acid as the positive control. Appropriate volumes of the sample and control solutions were accurately dispensed, and reagents were added according to the manufacturer’s instructions. Absorbance was measured at 734 nm [40].

$$\mathrm{ABTS}\cdot \;\mathrm{scavenging}\;\mathrm{rate}/\%=\frac{A_{\mathrm{Con}}-{\;A}_{\mathrm{Sample}}}{A_{\mathrm{Con}}}\times 100$$

Note: A_Con_ is the absorbance value measured after mixing reagents 1–5 with deionized water; A_Sample_ is the absorbance values measured after mixing reagents 1–5 with the sample.

### 4.5. Cell Culture and Experimental Treatment

The murine monocyte–macrophage leukemia cell line, RAW264.7, was maintained in Dulbecco’s Modified Eagle Medium (DMEM) supplemented with 10% fetal bovine serum (FBS) and 1% penicillin–streptomycin. The cultures were incubated at 37 °C in a humidified atmosphere containing 5% CO_2_. For experimental treatments, cells were pre-incubated with 20 μM of Hispidulin, Genkwanin, and Pectolinarigenin for 1 h. Subsequently, inflammatory responses were induced by the addition of lipopolysaccharide (LPS) at a final concentration of 1 μg/mL for a duration of 12–24 h, with the exception of the blank control group.

### 4.6. RT-qPCR Analysis

Total RNA was isolated from each treatment group and subjected to quantitative real-time PCR (qRT-PCR) analysis. GAPDH served as the endogenous internal control for normalization. The specific primer sequences utilized were as follows. GAPDH: Forward: 5′-AGGTCGGTGTGAACGGATTTG-3′; Reverse: 5′-GGGGTCGTTGATGGCAACA-3′. PTGS2: Forward: 5′-TGAGCAACTATTCCAAACCAGC-3′; Reverse: 5′-GCACGTAGTCTTCGATCACTATC-3′. Relative mRNA expression levels were calculated using the 2^- ΔΔCt^ method.

### 4.7. Data Statistical Analysis

Raw experimental data were initially processed in Microsoft Excel 2021. Figures were generated using Origin 2024, the Metware Cloud platform (https://cloud.metware.cn, accessed on 1 December 2025), and Cytoscape v3.9.1.

## 5. Conclusions

In this study, the antioxidant-active constituents and putative mechanisms of action of five *Ottelia* taxa (*Ottelia acuminata*, *O. acuminata* var. *jingxiensis*, *O. fengshanensis*, *O. guanyangensis*, and *O. alismoides*) were systematically identified and preliminarily validated through an integrated strategy combining metabolomics, network pharmacology, and in vitro antioxidant assays. Rivularin, tenaxin I, sinensetin, 8-methoxyapigenin, chrysoeriol, hispidulin, genkwanin, 5,2′-dihydroxy-7,8-dimethoxyflavone, kumatakenin, and pectolinarigenin were highlighted as key antioxidant-associated compounds across the five taxa. Network-based analyses suggested that PTGS1/2 and AR are major hub targets, and that antioxidant-related effects may be mediated, at least in part, through pathways such as PI3K–Akt signaling and EGFR tyrosine kinase inhibitor resistance. Collectively, these findings indicate that *Ottelia* species represent not only valuable ecological resources but also promising reservoirs of bioactive compounds with potential health applications, warranting further rigorous investigation.

## Figures and Tables

**Table 1:** The 25 chemical components that exhibit differences between every pair of species.

Compounds	Class I	Formula	Molecular Weight (Da)	CAS
Ent-16α,17-Dihydroxykauran-2-one	Terpenoids	C_20_H_32_O_3_	320.2351	-
2-[(4-hydroxyphenethyl)amino]ethane-1,1,2-triol O-Glucoside	Alkaloids	C_16_H_25_NO_9_	375.1529	-
Kaempferol-3-O-xylosyl(1→2)glucoside	Flavonoids	C_26_H_28_O_15_	580.1428	-
5,7-Dihydroxy-3,3′,4′,5′-tetramethoxyflavone	Flavonoids	C_19_H_18_O_8_	374.1002	14,585-04-7
Pectolinarigenin	Flavonoids	C_17_H_14_O_6_	314.079	520-12-7
Kaempferol-3-O-Apiosyl-(1→2)-Galactoside	Flavonoids	C_26_H_28_O_15_	580.1428	-
Kaempferol-3-O-sambubioside	Flavonoids	C_26_H_28_O_15_	580.1428	27,661-51-4
7-hydroxyaloin	Quinones	C_21_H_22_O_10_	434.1213	82,461-12-9
Naringenin-4′,7-dimethyl ether	Flavonoids	C_17_H_16_O_5_	300.0998	29,424-96-2
Gardenin D	Flavonoids	C_19_H_18_O_8_	374.1002	29,202-00-4
5,7-Dihydroxy-6,3′,4′,5′-tetramethoxyflavone (Arteanoflavone)	Flavonoids	C_19_H_18_O_8_	374.1002	68,710-17-8
1,4-Benzodioxin-6-propanol	Lignans and Coumarins	C_11_H_12_O_3_	192.0781	-
Demethoxysudachitin	Flavonoids	C_17_H_14_O_7_	330.074	4,323-80-2
2,6-Dimethoxy-4-hydroxyphenol-1-O-ß-D-glucopyranoside	Others	C_14_H_20_O_9_	332.1107	-
p-Hydroxypheny-β-D-allopyranoside	Others	C_12_H_16_O_7_	272.0896	-
Arbutin	Others	C_12_H_16_O_7_	272.0896	497-76-7
1-O-Caffeoyl-(6-O-glucosyl)-β-D-glucose	Phenolic acids	C_21_H_28_O_14_	504.1479	-
naringenin 5-glucoside	Flavonoids	C_21_H_22_O_10_	434.1213	-
1,5-O-dicaffeoyl-3-O-glucoside-quinic acid	Phenolic acids	C_31_H_34_O_17_	678.1802	-
Citraconic acid	Organic acids	C_5_H_6_O_4_	130.0266	498-23-7
Isochlorogenic acid C	Phenolic acids	C_25_H_24_O_12_	516.1268	57,378-72-0
Choerospondin	Flavonoids	C_21_H_22_O_10_	434.1213	81,202-36-0
Isochlorogenic acid A	Phenolic acids	C_25_H_24_O_12_	516.1268	2450-53-5
β-Hydroxy-(3,4-dihydroxyphenylethanolyl)-glucoside	Others	C_14_H_20_O_9_	332.111	-
Rhein-8-O-(6′-O-acetyl)glucoside	Quinones	C_23_H_20_O_12_	488.0955	-

**Table 2:** Seven MCODEs with closely related connections in the PPI network.

Cluster	Score (Density Nodes)	Nodes	Edges	Node IDs
1	15.111	19	136	TNF, RELA, MMP2, HIF1A, MAPK8, EGFR, BCL2, STAT3, SIRT1, CTNNB1, MMP9, ERBB2, PTGS2, EP300, GAPDH, JUN, SRC, ESR1, MAPK1
2	6	6	15	ALOX5, ALOX12, PLA2G4A, ALOX15, PTGS1, PLA2G2A
3	5	11	25	AR, APP, CDK2, GSK3B, PARP1, BCL2L1, MTOR, IGF1R, HSP90AA1, MAPK14, CASP3
4	4.4	6	11	CYP19A1, IDO1, CYP1B1, CYP1A2, CYP1A1, EPHX1
5	4	6	10	PTPN11, PTPN1, SYK, JAK2, KDR, FLT1
6	3	3	3	MMP1, MMP3, SERPINE1
7	3	3	3	ACE2, MME, DPP4

**Table 3:** Antioxidant core component–action target–pathway table.

Name	Type	Degree	Betweenness Centrality	Closeness Centrality
Rivularin	Component	22	0.0431733	0.2710280
Tenaxin I	Component	21	0.0295569	0.2663122
Sinensetin	Component	21	0.0575462	0.2798375
8-Methoxyapigenin	Component	18	0.0282008	0.2689117
Chrysoeriol	Component	18	0.0245711	0.2686494
Hispidulin	Component	16	0.0145355	0.2480864
Genkwanin	Component	14	0.0096068	0.2563983
5,2′-Dihydroxy-7,8-dimethoxyflavone	Component	14	0.0087965	0.2561599
Kumatakenin	Component	13	0.0084005	0.2561599
Pectolinarigenin	Component	12	0.00651711	0.2424109
PTGS2	Target	30	0.0868645	0.2821300
PTGS1	Target	20	0.0173976	0.2445628
AR	Target	19	0.0658289	0.2704958
DPP4	Target	18	0.0478600	0.2617577
Prostate cancer	Pathway	18	0.0287730	0.2571162
Pathways in cancer	Pathway	17	0.0251197	0.2556844
PI3K-Akt signaling pathway	Pathway	12	0.0061811	0.2304475
EGFR tyrosine kinase inhibitor resistance	Pathway	10	0.0031062	0.2237109

## Data Availability

The metabolomics data have been deposited in the MetaboLights (https://www.ebi.ac.uk/metabolights/, accessed on 26 April 2026) repository with the study identifier MTBLS14364.

## Supplementary Materials

The following supporting information can be downloaded at: https://www.mdpi.com/article/10.3390/ijms27094071/s1.
